# Clinicopathological findings in horses with a bi- or tripartite navicular bone

**DOI:** 10.1186/s12917-016-0698-4

**Published:** 2016-04-09

**Authors:** Ellen J. van der Zaag, Erik A. W. S. Weerts, Antoon J. M. van den Belt, Willem Back

**Affiliations:** Veterinary Clinic De Delta, Foppenpolder 1, NL-3155 EA Maasland, The Netherlands; Department of Pathobiology, Division Pathology, Faculty of Veterinary Medicine, Utrecht University, Yalelaan 1, NL-3584 CL Utrecht, The Netherlands; Department of Clinical Sciences of Companion Animals, Faculty of Veterinary Medicine, Utrecht University, Yalelaan 108, NL-3584 CM Utrecht, The Netherlands; Department of Equine Sciences, Faculty of Veterinary Medicine, Utrecht University, Yalelaan 112-114, NL-3584 CM Utrecht, The Netherlands; Department of Surgery and Anaesthesia of Domestic Animals, Faculty of Veterinary Medicine, Ghent University, Salisburylaan 133, B-9820 Merelbeke, Belgium

**Keywords:** Bi-tripartite navicular bone, Bi-tripartite sesamoids, Partitioning, Navicular fracture, Navicular pathology

## Abstract

**Background:**

Navicular bone partition is a rare condition reported in horses, which is during the evaluation of a lameness or prepurchase examination often misinterpreted for a parasagittal fracture. In this report, the clinicopathological findings of three cases of navicular bone partition are evaluated. The possible pathomechanisms underlying the condition are hypothesised, focusing on a potential origin of foetal vascular disturbance. This study is furthermore aiming at a clearer and earlier recognition of navicular bone partition, since this condition would finally predispose for a clinical lameness with a poor prognosis.

**Case presentations:**

Case 1 was a 10-year-old Belgian Warmblood gelding with a Grade 3/5 chronic, recurrent left-forelimb lameness that had persisted for 4 months. Perineural palmar digital nerve block of the distal foot abolished the lameness. Radiographic examination revealed a bipartite navicular bone in the left forelimb. Unfortunately, the animal was lost to follow-up. Case 2 was a 7-year-old Quarter Horse stallion with a Grade 3/5 recurrent right forelimb lameness that had persisted for 2 years. The lameness switched to the contralateral left forelimb with a palmar digital nerve block. Radiographic examination identified a tripartite navicular bone in both forelimbs. Pathological examination additionally revealed chronic degenerative changes of the cartilage and subchondral bone with marked cystic changes. Case 3 was a 5-year-old Dutch Warmblood gelding with a Grade 3/5 recurrent left hindlimb lameness that had persisted for 6 months. Owing to the uncooperative behaviour of the horse, only a combined peroneal and tibial nerve block could be performed, which abolished the lameness. Radiographic examination revealed a bipartite navicular bone in the left hindlimb. Pathological examination showed a navicular bipartition in the left hindlimb, with microscopic changes comparable to those evident in Case 2; additionally, cartilage indentations were also found in the navicular bones of the right front- and hindlimb at a similar location as the partition site in the left hindlimb.

**Conclusions:**

It is speculated that a navicular bone partition has a congenital origin and is caused by vascular disturbance during foetal development. This may lead to aberrant endochondral ossification or the formation of multiple ossification centres resulting in navicular bone partitioning. In the adult horse, chronic repetitive biomechanical challenges at the partition sites may induce local degenerative changes with subchondral cyst formation and thus would cause a gradually developing chronic lameness with a poor prognosis.

## Background

Bi- or tripartite development of the navicular bone is a rare condition in horses that is often a coincidental finding during the evaluation of lameness or at a prepurchase examination [3, 9, 15, 22]. Early recognition of the condition and distinction from its main differential diagnosis, a parasagittal fracture, is essential. Misinterpretation of radiographs can lead to disputes between the vendor, buyer, veterinarian and/or insurance company. The clinical appearance of navicular bone partition usually is a mild lameness, but affected horses may initially exhibit normal athletic function [9]. Its radiographic findings are characterized by rounded bone edges of the adjacent pieces of navicular bone, a wide radiolucent region at the partition site and often cystic development of adjacent subchondral bone, which are in contrast with the usual characteristics of a fracture [4, 5, 8, 9, 15, 18]. The veterinary literature on navicular bone partition is limited and pathological examination of the partite navicular bone has rarely been performed [18]. In the human literature, the condition has been reported more frequently, but to date it is merely considered a relatively uncommon anatomical variation [19]. Possible pathomechanisms underlying this condition mentioned, include the formation of multiple ossification centres and/or a disturbance in the blood supply, leading to incomplete ossification of the cartilaginous bone model [10, 11, 19].

In this article, the clinical, radiological and pathological examination of 3 horses with navicular bone partition will be presented and possible underlying pathogenetic mechanisms are discussed with a review of the existing relevant literature.

## Case presentation

Between 2008 and 2011, two adult Warmblood horses and one Quarter Horse were referred to the Equine Clinic of the Faculty of Veterinary Medicine at Utrecht University, The Netherlands, for the evaluation of a chronic, recurrent lameness of the left forelimb (LF), right forelimb (RF) and left hindlimb (LH), respectively. All horses were clinically evaluated i.e. the lameness was scored on a hard ground surface at trot on a straight line and on the lunge according to a modified AAEP lameness scale (Grade 0–5, separately for walk and trot). Palpation and percussion of the lame- and contralateral limb was performed and a flexion test of the distal limbs was scored as positive or negative. Local perineural analgesia was used to block the lameness and evaluated after 5–10 min. In two horses (Case 1 and 2) the lameness was blocked after a perineural analgesia of the distal foot using a palmar digital nerve (PDN) block. The third horse (Case 3) became sound after a peroneal and tibial nerve block. Bilateral radiographic evaluation of the navicular bone consisted of a dorsoproximal-palmarodistal oblique view, a lateromedial view and, if possible, a palmaro 45 ° proximal-palmarodistal oblique view. All three horses were diagnosed with a bi- or tripartite navicular bone in the lame limb; in one horse (Case 2) the condition was bilateral. Two horses (Cases 2 and 3) were euthanized because of the chronicity of the lameness and their poor prognosis, allowing a subsequent pathological examination (of both front feet in Case 2 and all four feet in Case 3). Case 1 was unavailable for follow-up.

### Case 1

A 10-year-old Belgian Warmblood gelding presented with recurrent LF lameness of gradual onset that had persisted for approximately 4 months. Clinical examination revealed a Grade 3/5 LF lameness on a straight line that became more severe on the left hand lunge. Palpation of the palmar aspect of the foot and percussion of the frog identified no abnormalities. The horse exhibited a positive response to a flexion test of the left distal forelimb and no response at the right distal forelimb. The lameness was abolished by a PDN block of the LF limb.

**Fig. 1:**
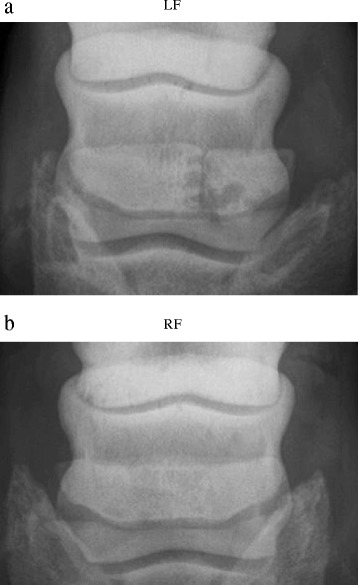
Case 1. Radiographic examination of a horse, dorsoproximal-palmarodistal oblique view. **a** Bipartite navicular bone conformation in the left forelimb. **b** Normal navicular bone conformation in the right forelimb

Radiographic examination of the LF in dorsopalmar view revealed bipartition of the navicular bone at the lateral wing with rounded adjacent edges. There was no clear formation of osteophytes and the distal border of the navicular bone had some small lucent zones. The lateral trabecular part of the navicular bone, however, showed several well-defined, radiolucent cystic areas adjacent to the radiolucent line (Fig. 1a). In the RF, in a similar dorsopalmar view of the navicular bone, a few radiolucent zones were visible on the distal border but there were no indications of partitioning or cystic development (Fig. 1b).

After the horse was diagnosed with a unilateral, bipartite navicular bone of the LF, the owner initially decided to further evaluate the use of the horse for hunting using orthopaedic shoeing, but finally the horse was lost to follow-up.

### Case 2

A 7-year-old Quarter Horse stallion presented with a recurrent RF lameness of gradual onset that had persisted for over 2 years. Clinical examination revealed a Grade 3/5 RF lameness comparable lame on both a straight line and on the right hand lunge. Palpation of the palmar pastern region of the RF elicited a mild pain response as-well as percussion of the frog. The horse exhibited a (slightly) positive response to a flexion test of the right forelimb and no response to the left forelimb. Using a PDN block of the distal RF the lameness switched to the LF limb.

**Fig. 2:**
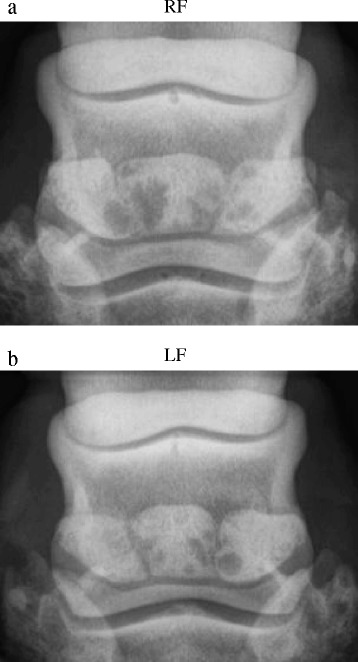
Case 2. Radiographic examination of a horse, dorsoproximal-palmarodistal oblique view with a tripartite navicular bone conformation in both forelimbs. **a** Right forelimb. **b** Left forelimb

Radiographic examination of both distal forelimbs in dorsopalmar view revealed that both navicular bones had extensive disseminated irregular radiolucent areas in the lateral and medial wings. The radiolucent lines, located at more or less one third of their length in the navicular bones, showed rounded corners at the proximal and distal edges. Both forefeet were diagnosed with a tripartite navicular bone (Fig. 2a, b). Due to the long history of lameness and poor prognosis, the horse was euthanized and both distal forelimbs became available for pathological examination.

**Fig. 3:**
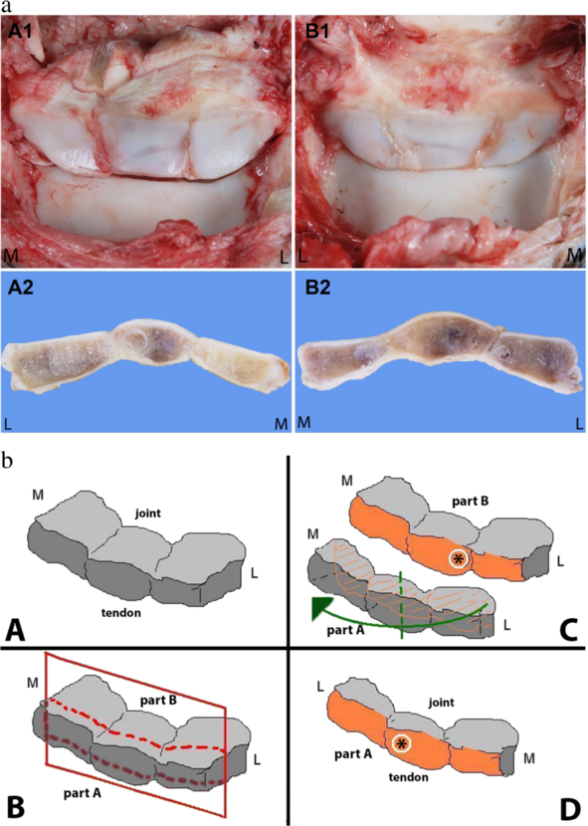
**a** Case 2. Macroscopic pathological findings of the LF (**A**) and RF (**B**) navicular bones (*panels 1*: distal interphalangeal joint articular surface, *panels 2*: transverse cut surface, formalin-fixed). Both navicular bones consist of three approximately same-sized bony pieces connected by fibrillary tissue. On the transverse cut surface of both bones, round variable-sized well-demarcated cystic structures can be appreciated near the connection sites. The articular surface is markedly irregular. L?=?lateral side, M?=?medial side. **b** Case 2. Transverse section of the navicular bones was made as shown in panel B (*red lines*), resulting in a front part A and a back part B. The Fig. 3: A2 and B2 are the front parts A of the bones after making the transverse sections and rotated 180°, as shown in panels C and D. The cystic change as situated in Fig. 3a: A2 is shown here as a *white circle (asterisk)*; L?=?lateral; M?=?medial

**Fig. 4:**
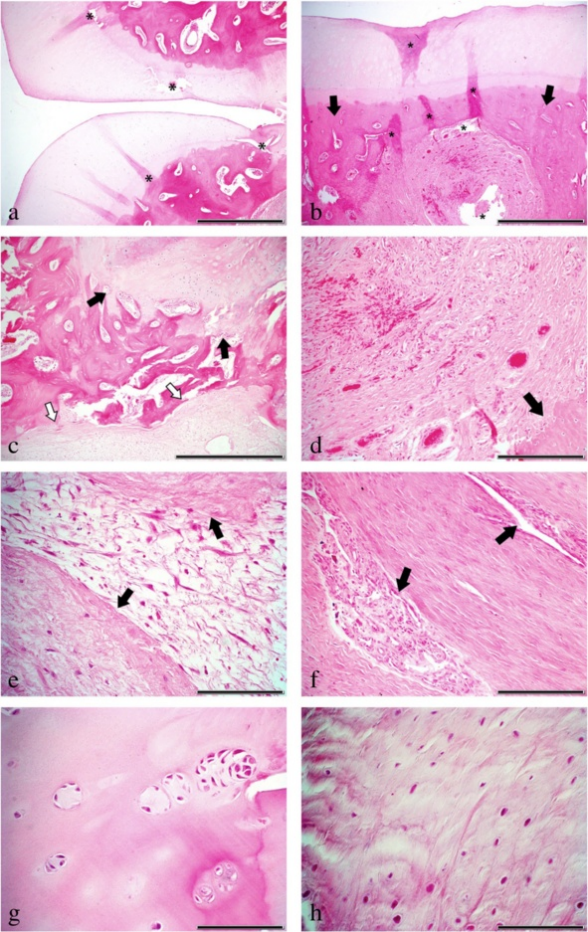
Combined histopathomorphological findings of Case 2 and 3, Haematoxylin and Eosin (H&E). **A** continuous smooth lining of cartilage, deviating into the bone at the partition’s connection site (bar = 1 mm). **B** cystic change of the subchondral bone with marked sclerosis of the adjacent bone (*arrows*) (bar = 1 mm). **C** retained cartilage cores (*black arrows*) at a connection site of one of the partitions near the outer concentric layer of a subchondral bone cyst (*white arrows*) (bar = 500 μm). **D** densely-packed highly vascular aspect of cysts, with adjacent sclerotic bone (*arrow*) (bar = 200 μm). **E** myxoid-like aspect of the cysts, surrounded by more densely-packed fibrous tissue (*arrows*) (bar = 100 μm). **F** island of tissue in the fibrous cystic wall resembling synovial membrane (*arrows*) (bar = 200 μm). **G** chondrone formation in the articular cartilage (bar = 100 μm). **H** fibrocartilaginous tissue at the partition’s connection sites (bar = 100 μm). **B**, **C**, **D**, **E**, **G** and **H** Case 2, LF navicular bone. **A** and **F**: Case 3, LH navicular bone; * = tissue processing artefacts

Pathological examination of the navicular bones of the RF and LF revealed similar abnormalities. Macroscopically, both bones appeared to be composed of three similar-sized pieces of bone, connected by small amounts of irregular slightly protruding pale yellow-brown to red fibrillar tissue, resulting in marked irregularity of the articular surface of the navicular bone to the middle phalanx at these connection sites. The cartilage of the middle third part of the bone lying in between these sites showed marked smooth indentation. On the transverse cut surface in the middle of the navicular bone, the connection sites consisted of fibrous tissue. Directly adjacent to these connection sites, the subchondral bone contained several variably-sized and sharply-demarcated grey-brown to white cystic areas. The synovial lining of the coffin joint and the adherent deep digital flexor tendon were slightly irregular, with yellow and red discoloration. The articular surfaces of the middle and distal phalanx of both coffin joints appeared smooth. Both coffin joints contained normal amounts of slightly viscous and slightly yellow synovial fluid (Fig. 3a). The transverse cut surface of the LF navicular bone was examined histopathomorphologically after fixation in 4 % formalin, decalcification, paraffin-embedding, sectioning at 4 μm, and Haematoxylin and Eosin (H&E) staining (Fig. 3b). The bone showed a continuous smooth lining of articular cartilage, deviating into the bone at the partition sites and culminating in extensive accumulations of fibrocartilaginous tissue in the centre of these sites. The adjacent articular cartilage at the coffin joint surface showed multifocal, irregular thickening and a tendency for mild chondrocyte clustering (chondrone formation). The adjacent subchondral bone appeared markedly sclerotic. The cystic lesions (lateral side of the middle third piece and the medial side of the lateral third piece) consisted of concentrically-layered accumulations of partly densely-arranged, moderately-cellular and highly-vascularized connective tissue and partly loosely-arranged myxoid-like connective tissue; no clear synovial epithelial lining was evident in the cysts. There were no signs of active inflammation or osteonecrosis. The macroscopically-described fibrillar tissue protruding at the partitions’ connection site surfaces could histologically not be traced back (Fig. 4).

### Case 3

A 5-year-old Dutch Warmblood gelding presented with recurrent LH lameness of gradual onset that had persisted for more than 6 months. Clinical examination revealed a Grade 3/5 LH lameness on a straight line and on the left hand lunge. Palpation of the palmar aspect of the foot and percussion of the frog identified no abnormalities. Owing to its dangerous, uncooperative behaviour, a flexion test and PDN block of the distal hindlimb could not be performed. Finally, a peroneal and tibial nerve block was performed on the lame LH, by which the lameness of the horse became abolished. Routine radiographs were made of the left hock and the left distal limb. After the horse was diagnosed with a LH bipartite navicular bone the contralateral navicular bone was also radiographically evaluated.

**Fig. 5:**
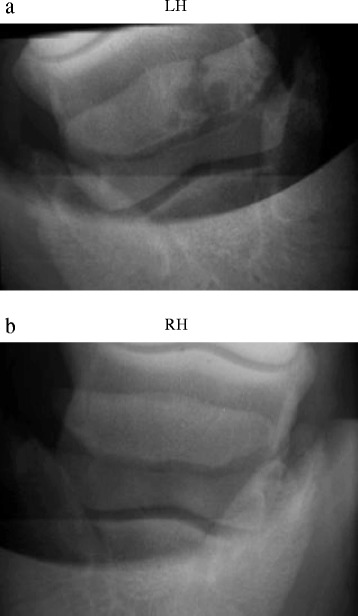
Case 3. Radiographic examination of a horse, dorsoproximal-palmarodistal oblique view. **a** Bipartite navicular bone confirmation in the left hindlimb. **b** Normal navicular bone confirmation in the right hindlimb

Radiographic examination of the LH revealed a wide, radiolucent line in the lateral wing of the navicular bone at one-third of its total length in a parasagittal plane, with rounded margins of the two osseous pieces and cystic changes in the adjacent subchondral bone, together indicative of bipartition; there were no clear findings consistent with degenerative joint disease (Fig. 5a). Further radiographic evaluation of the LH (fetlock and talocrural joint) and right hindlimb (RH) navicular bone (Fig. 5b) revealed no abnormalities. Due to the duration of the lameness, the depressed character change of the horse possibly due to the persistent pain, and the given poor prognosis, the horse was euthanized and a pathological examination was performed on all four feet.

**Fig. 6:**
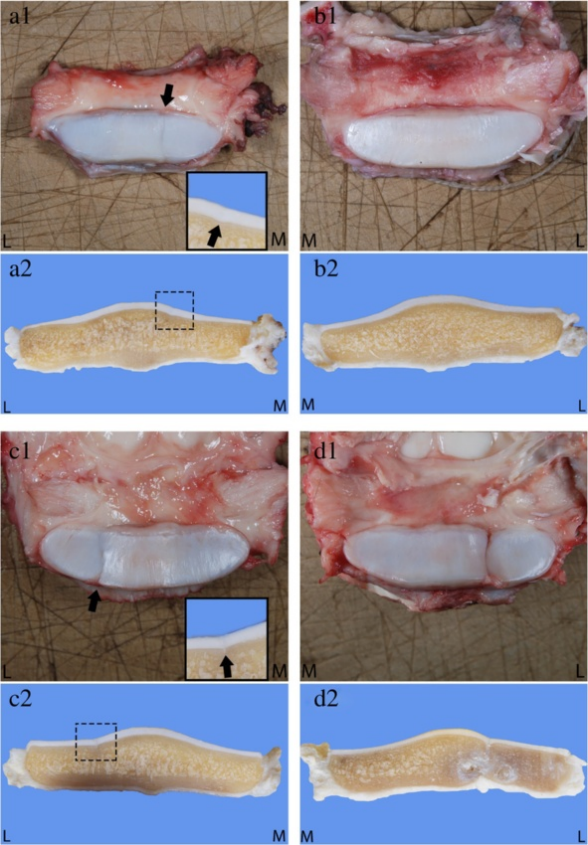
Case 3. Macroscopic pathological findings of the RF (**A**), LF (**B**), RH (**C**) and LH (**D**) navicular bones (*panels 1*: coffin joint articular surface, *panels 2*: transverse cut surface, formalin-fixed). The LH navicular bone shows marked sagittal indentation at its articular surface at the lateral side at one third of its length. On the transverse cut surface, cystic structures resembling those described in Case 2 can be appreciated at both sides of the connection site. The RF and RH navicular bones show only mild superficial sagittal indentation of the articular cartilage (picture insets), without changes of the subchondral bone; L = lateral side, M = medial side

Macroscopically, the LH navicular bone seemed to consist of two partly joined pieces with a clear sagittal indentation at the lateral side at one-third of its length, possessing smooth cartilaginous edges and causing marked focal irregularity of the articular surface to the middle phalanx. Contrasting with the partition sites in Case 2, the articular surface at this location did not contain any protruding irregular fibrillar tissue. On the transverse cut surface, adjacent to the edges of both bone parts, round variably-sized and sharply-demarcated white to grey-brown cystic structures were visible in the subchondral and trabecular bone. The RH and RF navicular bones showed only mild indentations in the lateral and the medial wings respectively of the articular cartilage at approximately one-third of the total length. The navicular bone of the LF appeared unremarkable. The articular surfaces of the middle and distal phalanx of all coffin joints were smooth. As described for Case 2, all coffin joints contained normal amounts of slightly viscous and slightly yellow synovial fluid (Fig. 6).

Histopathomorphologic examination of the LH navicular bone (fixation, decalcification, paraffin-embedding, slide thickness and staining identical to Case 2 revealed changes similar, though milder, to those in Case 2. There was mild irregular thickening of the otherwise continuous smooth lining of the cartilage, which deviated, similarly to Case 2, into the bone at the partition site. At this location, the navicular bone showed a similar accumulation of fibrocartilaginous tissue, few retained cartilage cores and sclerosis of the adjacent trabecular bone. Cystic lesions were of the same composition as in Case 2 and contained focally in the outer concentric layers near the connection site small tissue pieces resembling synovial lining (Fig. 4).

## Discussion

In both human medical and veterinary literature, navicular (distal sesamoid) bone partition is considered to be congenital [2–4, 11, 15, 21, 22]. In human medical literature, the most frequently suggested pathogenesis underlying sesamoid partition would involve the formation of multiple ossification centres within a single cartilage bone model, which fail to fuse [10, 11, 19], or the formation of extra aberrant ossification centres in addition to the normally present single cartilage bone model [19]. Regarding these hypothetic pathogenetic mechanisms, repetitive vascular micro-trauma [11] has been cited as an important aetiological factor, as it interferes with the ossification process by impairing blood, and therefore oxygen and nutrient, supply.

Typical features of a partitioned navicular bone, distinguishing it from parasagittal fractures, provided by this study are:

- a chronic, persistent lameness, often with a gradual onset;
- a correlation between relatively mild lameness and overt radiographic findings in the same limb(s);
- the possibility of multiple limbs involved;
- no substantial peri-articular remodelling;
- a uniform distribution of partition sites at approximately one-third of the length of the navicular bone; and
- a continuous, smooth lining of articular cartilage that deviates into the bone at the partition site.

Aetiologically, especially the last two characteristics, being rather distinctive anatomical features, seem to plead for abnormal congenital development of the involved navicular bones. The uniform distribution of the partition sites seen in all cases, notably in the case of tripartition when two partitions exist in the same bone evenly-spread along its total length simultaneously, is difficult to consider as being the result of a coincidental traumatic event leading to fractures. In addition, even if such simultaneous evenly-spread parallel fractures would be able to occur, clinical presentation would likely be very different with marked acute lameness dominating. Moreover, regarding the normal features of fracture repair and the normal regenerative properties of bone and cartilage, it is hardly thinkable that an uncomplicated smooth cartilaginous lining as seen at the partition sites in the described cases would develop at a site of a fracture. Whether the partitioning of distal or proximal sesamoid bones is a hereditary defect, is not that clear yet and beyond the scope of the findings presented in this study. In veterinary literature a ‘multiple ossification centre hypothesis’ is suggested as a possible pathomechanism underlying partitioning [3, 4, 8, 9]. Some authors otherwise regard this sesamoid bone partitioning as a mild form of polydactyly [2, 21]. If navicular bone partitioning is an expression of polydactyly, however, one might expect further partitioning of also of the more proximal bones and surrounding structures (e.g. tendons) distal to the involved navicular bone. The authors of this paper found no evidence of this in their case series and can therefore would rather not support polydactyly as the underlying mechanism of navicular bone partition.

**Fig. 7:**
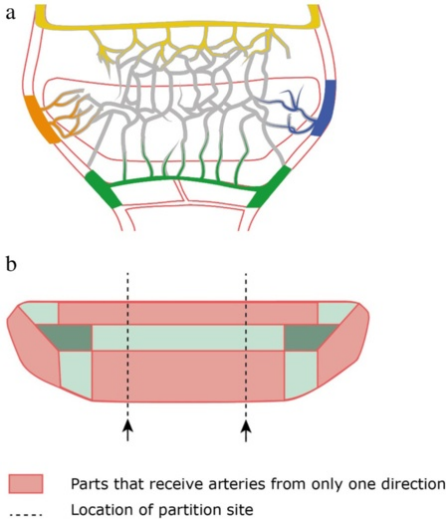
**a** Schematic drawings of the arterial supply of the navicular bone (modified with permission from Rijkenhuizen et al. [16]). Arteries entering from four different directions illustrated in colours. **b** Diagram of the suggested arterial anatomy of the navicular bone (modified with permission from Rijkenhuizen et al. [16]). The red coloured areas indicate the parts of the navicular bone that receive arteries from only one direction. The light green coloured areas receive arteries from two directions and the dark green coloured areas from three directions

During fetal development, the blood supply to the navicular bone takes two routes from day 125 of gestation [17]. One supply is temporarily situated in the superficial layer of the fibrocartilage; the other supply is the one becoming permanent in the adult horse [16], in which arteries enter the navicular bone from four different directions (Fig. 7a). The temporary secondary circulation gradually disappears after 270 days of gestation when the first ossification centres appear. A disruption of either of these vascular networks might trigger aberrant ossification, although the temporary vessels are less likely to be of influence as they do not penetrate the navicular bone itself. Vascular micro-trauma or intra-articular blood vessel compression due to e.g. unusual positioning of the foetus in utero may lead to hypoxia and result in defective endochondral ossification, eventually causing partitioning of the navicular bone. In a similar way, regarding the pathogenesis of osteochondrosis during bone and joint growth, failure of the blood supply is considered responsible for the defective transformation of cartilage into bone [13, 14, 23]. In a study of foals [13, 14], early lesions of osteochondrosis in the distal tibia occurred secondary to failure of the blood supply of the physeal plate, leading to ischaemic necrosis and locally preventing normal ossification. Moreover, osteochondrotic lesions are formed in a limited time window, namely the period when the epiphyseal cartilage is supplied by vulnerable blood vessels [23]. Our hypothesis is based on the same idea that disturbance at a specific moment during gestation, when angiogenesis in the cartilage navicular bone model occurs [17], can initiate the partitioning process.

Partitions of the navicular bone seem to be consistently located at approximately one-third of the length of the bone in the parasagittal plane in the lateral and/or medial wing. Certain parts of the navicular bone receive arteries from only one side, whereas other parts receive arteries from more than one direction [16, 17]. The parts supplied by only one artery are likely more susceptible to ischemia due to vascular disturbance, which during foetal development might predispose these areas for partitioning (Fig. 7b).

The histological changes found at partition sites can be briefly summarized as features of degeneration. A suitable explanation for these changes is that the partition sites are subjected to abnormal pressure through micro-movement of the bone segments during life [6, 12], again (as with the hypothesized pathogenesis of the partitioning process in utero) compromising local circulation and leading to ischaemic necrosis. Within the rather non-specific spectrum of degenerative lesions of bone, subchondral cysts are chronic lesions that likely develop from necrotic cartilaginous fragments retained in the subchondral bone, focal necrosis of the subchondral bone itself or as a result of synovial fluid being forced into the subchondral bone via defects in the articular cartilage [1]. This is seen as a sequel of osteochondrosis [1], in the course of navicular disease [7] and also after trauma to the articular surface [1, 20]. It is unclear whether the cysts in the presented cases have been formed via necrotic retained cartilaginous fragments (which are a finding in Case 3) or from the subchondral bone itself. Nevertheless, because of their known gradual development during various chronic joint disease, subchondral cysts are more likely to co-occur with partitioning and its subsequent degenerative features than with an acutely occurring fracture; therefore, they might be of differential diagnostic significance during radiological examination.

In our case series there was a clear age difference (5-7-10 years) in occurrence of clinical and radiological symptoms, which is consistent with other reported cases [2, 3, 18]. All horses had similar workload and were regularly ridden; only one horse had a history of sliding in the pasture. Apparently, the onset of specific clinical and radiological features is not directly age-related, but indirectly related to the severity of the joint pressure imbalances and therefore subsequently the amount of time needed for the bone parts to develop the described degenerative features presumably as a result of these imbalances.

In summary, when we compare our case findings, the final lameness in the partitioned navicular bone seems to be mainly linked to bone degeneration with cystic changes. Case 1 showed cyst-like abnormalities during radiographic examination in only the lame limb; in this case, lameness was abolished after a PDN block. In Case 2, the involved horse showed cystic changes in the navicular bones of both front legs; the horse became lame in the contralateral limb after PDN block. In Case 3, after the lame limb was blocked (unfortunately the diagnosis could not definitively be confirmed by a PDN block), lameness did not switch to the contralateral limb, which is in accordance with the radiological and pathological appearance of the bone, showing no overt abnormalities.

## Conclusions

Navicular bone partitioning likely has a congenital origin and can possibly be explained as the result of articular vascular disturbance in utero, causing bone abnormalities during foetal bone formation by endochondral ossification. Its clinical appearance is a relatively mild lameness of gradual onset. The partitioning of the bone is already overt at birth and moreover due to the biomechanical challenges at the partioning site during life this induces a progressing focal (cystic) degeneration of the navicular bone causing a gradually developing lameness. The cysts and other characteristic radiographic findings, which include the presence of two or three clearly separate pieces at approximately one-third of the length of the bone with rounded margins and minimal peri-articular remodelling, are important features distinguishing navicular bone partitioning from its main differential diagnosis, the parasagittal fracture.

The horses described in this study already showed clinical signs consistent with the abnormal radiographs and pathological findings. They seem to have exhibited the chronic stage of the disease, mainly reflecting secondary degenerative processes. Detection of the partitioning process earlier in life may result in a better understanding of the progression of this condition, thus leading to a better insight in the disease. Earlier recognition by the veterinarian might prevent occurrence of prepurchase issues and furthermore save time and money otherwise becoming invested in horses suffering from an abnormality with finally a poor prognosis.

### Ethics approval and consent to participate

In all admitted cases their owners gave their written informed consent to participate by means of signing our official client acceptance form.

### Consent for publication

In all admitted cases their owners gave their written informed consent for publication by means of signing our official client acceptance form.

### Availability of data and materials

The data supporting our findings are contained within the manuscript.

## Abbreviations

LF: left forelimb
LH: left hindlimb
PDN: palmar digital nerve
RF: right forelimb
RH: right hindlimb
